# hnRNP C modulates MERS-CoV and SARS-CoV-2 replication by governing the expression of a subset of circRNAs and cognitive mRNAs

**DOI:** 10.1080/22221751.2022.2032372

**Published:** 2022-02-10

**Authors:** Xi Zhang, Hin Chu, Kenn Ka-Heng Chik, Lei Wen, Huiping Shuai, Dong Yang, Yixin Wang, Yuxin Hou, Terrence Tsz-Tai Yuen, Jian-Piao Cai, Shuofeng Yuan, Feifei Yin, Kwok-Yung Yuen, Jasper Fuk-Woo Chan

**Affiliations:** a State Key Laboratory of Emerging Infectious Diseases, Carol Yu Centre for Infection, Department of Microbiology, Li Ka Shing Faculty of Medicine, The University of Hong Kong, Pokfulam, People’s Republic of China; b Key Laboratory of Translational Tropical Medicine of Ministry of Education, Hainan Medical University, Haikou, People’s Republic of China; c Academician Workstation of Hainan Province, Hainan Medical University, Haikou, People’s Republic of China; d Hainan Medical University-The University of Hong Kong Joint Laboratory of Tropical Infectious Diseases, Hainan Medical University, Haikou, People’s Republic of China; e Department of Clinical Microbiology and Infection Control, The University of Hong Kong-Shenzhen Hospital, Shenzhen, People’s Republic of China; f Department of Microbiology, Queen Mary Hospital, Pokfulam, Hong Kong Special Administrative Region, People’s Republic of China; g Centre for Virology, Vaccinology and Therapeutics, Hong Kong Science and Technology Park, Hong Kong Special Administrative Region, People’s Republic of China

**Keywords:** Coronavirus, circRNA, hnRNP C, mRNA, RNA binding protein

## Abstract

Host circular RNAs (circRNAs) play critical roles in the pathogenesis of viral infections. However, how viruses modulate the biogenesis of host proviral circRNAs to facilitate their replication remains unclear. We have recently shown that Middle East respiratory syndrome coronavirus (MERS-CoV) infection increases co-expression of circRNAs and their cognate messenger RNAs (mRNAs), possibly by hijacking specific host RNA binding proteins (RBPs). In this study, we systemically analysed the interactions between the representative circRNA–mRNA pairs upregulated upon MERS-CoV infection and host RBPs. Our analysis identified heterogeneous nuclear ribonucleoprotein C (hnRNP C) as a key host factor that governed the expression of numerous MERS-CoV-perturbed circRNAs, including hsa_circ_0002846, hsa_circ_0002061, and hsa_circ_0004445. RNA immunoprecipitation assay showed that hnRNP C could bind physically to these circRNAs. Specific knockdown of hnRNP C by small interfering RNA significantly (*P* < 0.05 to *P* < 0.0001) suppressed MERS-CoV replication in human lung adenocarcinoma (Calu-3) and human small airway epithelial (HSAEC) cells. Both MERS-CoV and severe acute respiratory syndrome coronavirus 2 (SARS-CoV-2) infection increased the total and phosphorylated forms of hnRNP C to activate the downstream CRK-mTOR pathway. Treatment of MERS-CoV- (IC_50_: 0.618 µM) or SARS-CoV-2-infected (IC_50_: 1.233 µM) Calu-3 cells with the mTOR inhibitor OSI-027 resulted in significantly reduced viral loads. Collectively, our study identified hnRNP C as a key regulator of MERS-CoV-perturbed circRNAs and their cognate mRNAs, and the potential of targeting hnRNP C-related signalling pathways as an anticoronaviral strategy.

## Introduction

Eukaryotic splicing machineries tightly modulate the outputs of pre-messenger RNAs (mRNAs) in mammalian cells by initiating canonical splicing to produce linear mRNAs or back-splicing to produce circular RNAs (circRNAs) [1, 2]. Human circRNAs sponge multiple RNA transcripts and RNA-binding proteins (RBPs) to regulate gene expression. On the other hand, circRNA expression could also in turn be regulated by RBPs recruited in or around the exons, which generates circRNAs during the splicing process [3, 4]. To date, most splicing factors reported as circRNA regulators have been identified to function via direct binding mediated by matched motifs on specific circRNA sequences, including muscleblind [5], Quaking homolog, KH domain RNA binding (QKI) [6], complexes of multiple heterogeneous nuclear ribonucleoproteins (HNRNPs) and serine–arginine (SR) proteins [7], Fused in Sarcoma (FUS) protein [8], and heterogeneous nuclear ribonucleoprotein C (hnRNP C) [9].

The roles of host circRNAs in viral infections have been increasingly recognized in recent years [10, 11]. However, how viruses modulate the biogenesis of host proviral circRNAs to facilitate their replication remains unclear. Given the profound impact of RBPs on the splicing process and circRNA biogenesis, we hypothesize that viruses could hijack RBPs during pre-mRNA splicing, therefore manipulating the expression of certain circRNAs and cognate mRNAs for efficient replication. Extending from our recent report on the competing endogenous RNA network profiling of Middle East respiratory syndrome coronavirus (MERS-CoV) infection in human pulmonary Calu-3 cells [12], here we systemically analysed MERS-CoV-altered RBPs that might function as splicing factors. Our data identified hnRNP C as a host dependency factor governing the expression of multiple MERS-CoV-perturbed circRNAs, including hsa_circ_0002846, hsa_circ_0002061, and hsa_circ_0004445, as well as their cognate linear mRNAs. Ablation of hnRNP C more potently suppressed MERS-CoV replication in Calu-3 cells and primary human small airway epithelial cells (HSAECs) compared with individual silencing hsa_circ_0002846, hsa_circ_0002061, and hsa_circ_0004445. In exploring the applicability of hnRNP C as a broad-spectrum antiviral target, we observed a strong relevance of the hnRNP C-mediated circRNA expression machinery with the pathogenesis of other human-pathogenic coronaviruses, including SARS-CoV and SARS-CoV-2. The expression of one of the mutual downstream gene targets of hnRNP C, CT-10 regulator of kinase (CRK), was suppressed upon hnRNP C knockdown during the infection of MERS-CoV and SARS-CoV-2, which limited the phosphorylation of mTOR and reduced viral replication. Treatment of MERS-CoV-infected and SARS-CoV-2-infected cells with the mTOR inhibitor OSI-027 resulted in significantly reduced viral loads. Collectively, our study provided novel mechanistic insights on the virus-hijacked splicing machinery and a new layer of virus-host interactions involving proviral circRNAs, cognate mRNAs and RBPs in the context of coronavirus infections. Specific inhibition of the critical splicing factor hnRNP C or the downstream mTOR significantly reduced replication of MERS-CoV and SARS-CoV-2.

## Materials and methods

### Viruses and cells

MERS-CoV (strain HCoV-EMC/2012) was kindly provided by Ron Fouchier (Erasmus Medical Center, Rotterdam, the Netherlands). Clinical isolates of SARS-CoV (GZ50) and SARS-CoV-2 HKU-001a were available at the Department of Microbiology at The University of Hong Kong as we previously described [13, 14]. Calu-3 (human lung adenocarcinoma) cells were maintained in Dulbecco's Modified Eagle Medium/F12 (DMEM/F12) supplemented with 10% heat-inactivated fetal bovine serum (FBS), 100 U/ml penicillin and 100 µg/ml streptomycin as previously described [15]. Primary HSAECs and the complete growth medium for cell culture were obtained from ATCC®.

### Antibodies

The in-house guinea pig anti-MERS-CoV nucleocapsid protein (NP) serum and negative control guinea pig serum were used for MERS-CoV NP detection in flow cytometry experiments as we previously described [16]. Mouse anti-hnRNP C antibody (clone 4F4, IgG1) (Santa Cruz Biotechnology, sc-32308) was used in flow cytometry, Western blot, and RNA immunoprecipitation (RIP) assay. Anti-eIF4EBP1 (phosphor T36) antibody (Abcam, ab47365), anti-phospho-hnRNP C (Ser260) antibody (Thermo Fisher Scientific, Waltham, MA, USA, PA5-38589), anti-mTOR antibody (Abcam, ab134903), anti-mTOR (phospho S2481) antibody (Abcam, ab137133), anti-CRK antibody (Abcam, ab133581), anti-β-actin antibody (Sigma, A5441) were used in western blot analyses as primary antibodies for the detection of each respective target protein. Goat anti-rabbit IgG (H + L) HRP antibody (Thermo Fisher Scientific, 656120), and goat anti-mouse IgG (H + L) HRP antibody (Thermo Fisher Scientific, 626520) were used as secondary antibodies in the western blot. Rabbit IgG (Abcam, ab37415) and mouse IgG1 (Abcam, ab81032) were used as isotype control antibodies.

### Integrated analysis of MERS-CoV-perturbed splicing factors, circRNAs and mRNAs

Firstly, differential expression (DE) analysis was performed to identify DE splicing factors, DE circRNAs and their cognately expressed mRNAs according to our previous methods [12]. Then, Pearson correlation coefficients (PCCs) and correlation *P* values (cPs) were calculated based on the expression level of these DE circRNAs and mRNAs at three time points. CircRNAs and their cognate mRNAs that stably expressed [average expression >10 transcripts per million (TPM)/fragments per kb for a million reads (FPKM)], both significantly upregulated (fold change ≥2 and adjust *P* value <0.05) and positively co-expressed (PCC > 0.7, c*P* < 0.05) were filtered out as potential MERS-CoV pathogenic circRNA-mRNA pairs for further study. To predict the splicing factors regulating their expression, correlation analysis between DE splicing factors and DE circRNAs or mRNAs identified above was performed simultaneously by the same method. At the same time, RBPmap (http://rbpmap.technion.ac.il/) database was used to predict the potential binding motifs between circRNAs and splicing factors to furtherly filter the dominant functional splicing factors.

### RNA extraction and quantitative reverse transcription-polymerase chain reaction (RT-qPCR)

The total RNA was extracted using MagJET RNA Kit (Thermo Fisher Scientific, K2731) following the manufacturer's instructions. The extracted RNA was subjected to one-step reverse transcription quantitative polymerase chain reaction (RT-qPCR) with QuantiNova SYBR Green RT–PCR Kit (Qiagen, 208154). Divergent primers for the selected circRNAs were designed through CircInteractome (https://circinteractome.nia.nih.gov/) (Supplementary Table 1). Fold changes of circRNA and host gene expression were calculated through the comparative CT (2^− ΔΔCT^) method and normalized with the expression of housekeeping gene GAPDH. For viral load quantification, RT-qPCR assays were performed for MERS-CoV, SARS-CoV, and SARS-CoV-2 as we described previously [17–19]. The MERS-CoV, SARS-CoV and SARS-CoV-2 standard plasmids under 10-fold serial dilutions equivalent to 10^3^–10^7^ copies per reaction were used to generate the calibration standard curve [20].

#### RIP

Calu-3 cells (5 × 10^6^) were inoculated with a multiplicity of infection (MOI) of 0.1 MERS-CoV at 37°C or under mock infection. At 24 hours post-infection (hpi), mock-infected and MERS-CoV-infected Calu-3 cells were harvested in lysis buffer for RIP without cross-linking using Pierce Immunoprecipitation Kit (Thermo Fisher Scientific, 26147) according to the manufacturer's protocol. To be more specific, the clear lysate of each sample was incubated with Protein A/G Plus Agarose beads conjugated with 10 μg mouse isotype-matched control IgG1 antibody or mouse anti-hnRNP C antibody (clone 4F4, IgG1) (sc-32308, Santa Cruz Biotechnology, Inc., Dallas, TX, USA) at 4°C overnight, respectively. Then beads were washed using washing buffer and the immunoprecipitated RNA-protein complexes were eluted for the detection of hnRNP C by Western blot, or were subjected to total RNA extraction and RT-qPCR for the detection of target circRNAs.

### siRNA knockdown and infection

Customized siRNAs targeting circRNAs, SMARTpool ON-TARGETplus human hnRNP C siRNA, SMARTpool ON-TARGETplus human CRK siRNA and ON-TARGETplus non-targeting pool siRNA were synthesized by Dharmacon (Lafayette, CO, USA). The detailed sequences of the customized siRNAs used in this study were listed in Supplementary Table 1. Calu-3 cells or HSAEC were transfected with 70 nM siRNA using Lipofectamine RNAiMAX (Thermo Fisher Scientific) twice over two consecutive days as previously described [21, 22]. At 24 h after the second transfection, the cells were challenged with MERS-CoV (MOI = 0.1), SARS-CoV (MOI = 1.0), or SARS-CoV-2 (MOI = 1.0). The inoculum was removed after 1 h at 37°C. The knockdown efficiency of selected circRNAs or genes was assessed by RT-qPCR or Western blot in parallel. At 24 hpi, the cells were harvested for further analysis.

### Flow cytometry

Calu-3 cells consecutively transfected with either scramble siRNAs or hnRNP C siRNAs were challenged with MERS-CoV at 1.0 MOI and harvested at 24 hpi for flow cytometry. Sample preparation and immunostaining were performed following standard procedures as we previously described [20, 23]. Specifically, cells were detached with 10 mM EDTA in PBS and fixed in 4% paraformaldehyde. 0.1% Triton X-100 in PBS was used for cell permeabilization and intracellular staining. Endogenous expression of MERS-CoV NP and hnRNP C were detected by co-immunostaining using in-house guinea pig anti-MERS-CoV NP serum and anti-hnRNP C antibody. Negative guinea pig serum and mouse IgG1 were served as Isotype controls. Alexa Fluor 647 goat anti-guinea pig, and Alexa Fluor 488 goat anti-mouse were used as secondary antibodies. The flow cytometry was performed using a BD FACSCanto II flow cytometer (BD Biosciences) and data was analysed using FlowJo vX (Tree Star). The experiment was repeated for three times for the quantification of the MERS-CoV NP positive rate.

### CC_50_ and IC_50_ determination of mTOR inhibitor in Calu-3 cells

The selective mTOR inhibitor OSI-027 was purchased from Selleckchem (Houston, TX, USA) and was titrated in different concentrations (0-40 μM) for pre-treatment, infection and post-infection incubation. After 1 h of drug pre-treatment, Calu-3 cells were inoculated with 0.1 MOI MERS-CoV or 1.0 MOI SARS-CoV-2 for half-maximal inhibitory concentration (IC_50_) assay. The inoculum was removed after 1 h of virus absorption and replaced with various concentrations of drug-containing medium. Cell lysates were harvested at 24 hpi and subjected to RNA extraction and RT-qPCR to assess viral gene copies. The IC_50_ was calculated using GraphPad Prism as we previously described [24, 25]. In parallel, mock-infected Calu-3 cells were treated with each titrated OSI-027 medium for 24 h to examine the 50% cytotoxic concentrations (CC_50_). The cell viability was evaluated with CellTiterGlo® as described before [26–28]. All experiments were performed in triplicates for the calculations of CC_50_ and IC_50_.

### Statistical analyses

Statistical comparisons between different groups were performed by one-way ANOVA, two-way ANOVA, or Student's t-test as indicated in the figure legends by GraphPad Prism (GraphPad Software). *P*-value of less than 0.05 was considered to be significant.

## Results

### MERS-CoV-infected human lung epithelial cells demonstrate a unique co-expression pattern of circRNA-cognate mRNA

**Figure 1.**
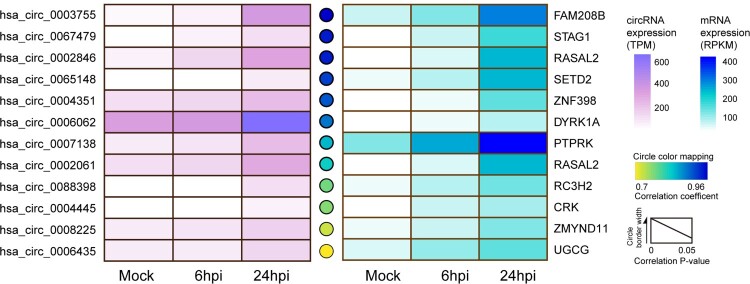
Identification of the representative circRNA-cognate mRNA pairs perturbed in MERS-CoV infection. The expression kinetics of the representative circRNA–cognate mRNA pairs perturbed in MERS-CoV infection. The selected circRNA–mRNA pairs were significantly upregulated during MERS-CoV infection (fold change ≥2 and adjust *P* value <0.05) and positively co-expressed (Pearson correlation coefficient >0.7, correlation *P* values <0.05). Heatmaps were separately drawn based on the expression intensity of each circRNA (left panel) or mRNA (right panel) at different time points. Circles between the two heatmap represented the correlation between each circRNA and its cognate mRNAs. Deeper colour density indicated stronger positive correlations. The circle border width was mapped according to the correlation *P* value.

The correlation between the expression of circRNAs and their cognate mRNAs remains incompletely understood [1, 2, 29–31]. We have previously shown that the majority (91.9%, 25752/28025) of the circRNAs detected in mock-infected Calu-3 cells showed no significant co-expression pattern with their cognate mRNAs, while upon MERS-CoV infection, the dynamic expression of over 60% (1112/1815) of DE circRNAs either positively or negatively correlated with their cognate mRNAs (Supplementary Figure 1), with a dominant proportion on positive correlation pairs (82.9%, 922/1112) [12]. These previous data was submitted to the GEO database (http://www.ncbi.nlm.nih.gov/geo/) under the accession number GSE139516. The expressions of the 12 most strongly upregulated and positively-correlated circRNA-cognate mRNA pairs during MERS-CoV infection were shown in Figure 1. These results suggested a novel regulatory mechanism of circRNA biogenesis induced by MERS-CoV infection in which each of the significantly triggered circRNA-mRNA pairs with positive correlation originated from the activation of transcription of the same genes.

Incorrect *in silico* prediction of the back-splicing sites on individual circRNAs may lead to false prediction of circRNA expression and thus over- or underestimation of correlation between circRNAs and their cognate mRNAs [32]. Thus, we performed *in vitro* validation by first synthesizing divergent and convergent RT-qPCR primers to examine the expression of the circRNAs and mRNAs (Supplementary Table 1). A total of 11 out of 12 circRNAs and their cognate mRNAs (10/11) could be consistently detected with or without MERS-CoV infection as shown in Supplementary Figure 2. The expression of most of the detectable RNAs was consistent with the results of RNA sequencing, except for hsa_circ_0004351 and its cognate mRNA ZNF398, which were not upregulated upon MERS-CoV infection. Finally, 10 experimentally validated circRNA candidates and their cognate mRNAs were selected for subsequent analysis, namely hsa_circ_0003755, hsa_circ_0067479, hsa_circ_0002846, hsa_circ_0006062, hsa_circ_0007138, hsa_circ_0002061, hsa_circ_0088398, hsa_circ_0004445, hsa_circ_0008225, and hsa_circ_0006435.

### Identification of splicing factors that potentially regulate the generation of MERS-CoV-perturbed circRNA-mRNA pairs

**Figure 2.**
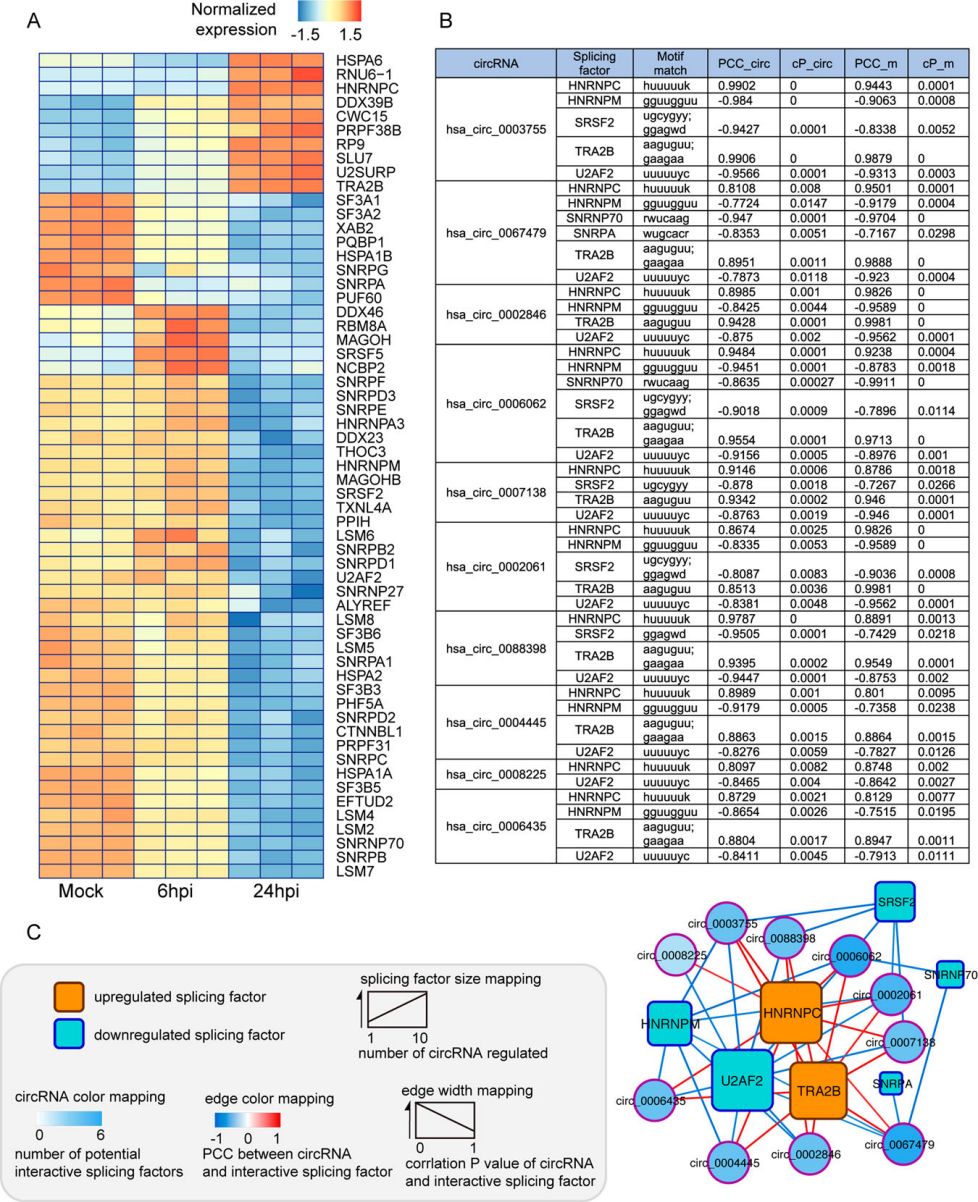
*In silico* analysis identifies splicing factors potentially regulating the generation of MERS-CoV-perturbed circRNA-mRNA pairs. (A) Heatmap presentation of MERS-CoV-perturbed differentially expressed (DE) splicing factors. The splicing factors were under hierarchical clustering and coloured by its normalized intensity scale [log_2_ (expression values in transcripts per million)]. (B) Candidate splicing factors potentially governing the expression of the representative circRNA-cognate mRNA pairs perturbed in MERS-CoV infection. The predicted binding motifs between each circRNA and splicing factor, Pearson correlation coefficients between each circRNA and splicing factor (PCC_circ) with its corresponding correlation *P* value (cP_circ), and Pearson correlation coefficients between each mRNA and splicing factor (PCC_m) with its corresponding correlation *P* value (cP_m) were listed. (C) Network demonstrating the interactions among MERS-CoV-perturbed circRNAs and splicing factors. Splicing factors and circRNAs were represented by rectangular and circular nodes, respectively. The general expression trend of each splicing factor during MERS-CoV infection was distinguished by filling colour (orange: up-regulated; blue: down-regulated). The size of splicing factors was proportional to the number of circRNAs potentially under their regulation. Among the seven splicing factors, HNRNPC and U2AF2 stood out to be the largest ones as they were predicted to regulate the highest number of circRNAs (10 circRNAs) over other candidate splicing factors. The filling colour density of each circRNA was mapped according to the number of its potential interactive splicing factors. Edge colour and thickness were proportionally correlated with the correlation coefficiency and correlation *P* value between each circRNA and splicing factor, respectively.

The co-expression of circRNAs and their cognate mRNAs suggested a unique regulatory machinery activated upon MERS-CoV infection. As many RBPs predominantly function as splicing factors on circRNA production [5–8], we postulated that the expression of MERS-CoV-perturbed circRNA-mRNA pairs might be a consequence of the modulation exerted by RBPs on pre-mRNA splicing. To test our hypothesis, we conducted an in-depth analysis of the expression pattern of DE genes in MERS-CoV-infected Calu-3 cells that we previously identified [12] and selected those recorded as RBPs in the Spliceosome pathway in the KEGG database (Figure 2A). RBPs assemble with RNA to form ribonucleoproteins (RNPs) through RNA-binding domains (RBDs), including the RNA recognition motif, K-homology domain, DEAD box helicase domain, dsRNA motif, zinc-fingers, and others [33]. Most RBPs involve in circRNA biogenesis via binding to specific sites of the circRNA [5–8, 34]. To identify the specific splicing factors potentially regulating the biogenesis of MERS-CoV-perturbed circRNAs, we predicted the interactions between the 10 MERS-CoV-perturbed circRNAs (Figure 1B) and the DE splicing factors (Figure 2A) by searching the RNA binding motifs through RBPmap database (http://rbpmap.technion.ac.il/). Based on the assumption that this regulation should be tightly modulated by virus during infection, we further filtered the splicing factors by individually calculating their Pearson Correlation Coefficient (PCC) and correlation *P*-value (cP) with the selected circRNAs and cognate mRNAs, and then selected those holding dual significant correlations (|PCC|>0.7, c*P* < 0.05) with both circRNAs and mRNAs for further verification. As a result, 7 splicing factors [up-regulated: HNRNPC and transformer-2 protein homolog beta (TRA2B); down-regulated: HNRNPM, serine/arginine-rich splicing factor 2 (SRSF2), U1 small nuclear ribonucleoprotein 70 kDa (SNRNP70), U1 small nuclear ribonucleoprotein A (SNRPA), and splicing factor U2AF 65 kDa subunit (U2AF2)] were identified (Figure 2B). Among the identified splicing factors, HNRNPC and U2AF2 each showed broad associations with the generation of 10 circRNAs and their cognate mRNAs, which indicated their importance in our hypothesized transcription regulatory response (Figure 2C). Moreover, the translational product of gene HNRNPC, known as a heterogeneous nuclear ribonucleoprotein hnRNP C, was the only splicing factor among the seven identified RBPs with characterized potency on circRNA regulation [9, 35] and was significantly upregulated upon MERS-CoV infection. We therefore selected hnRNP C for further investigation as a potential splicing regulator during MERS-CoV infection.

### hnRNP C coordinates the expression of MERS-CoV-perturbed circRNA and their cognate mRNAs

**Figure 3.**
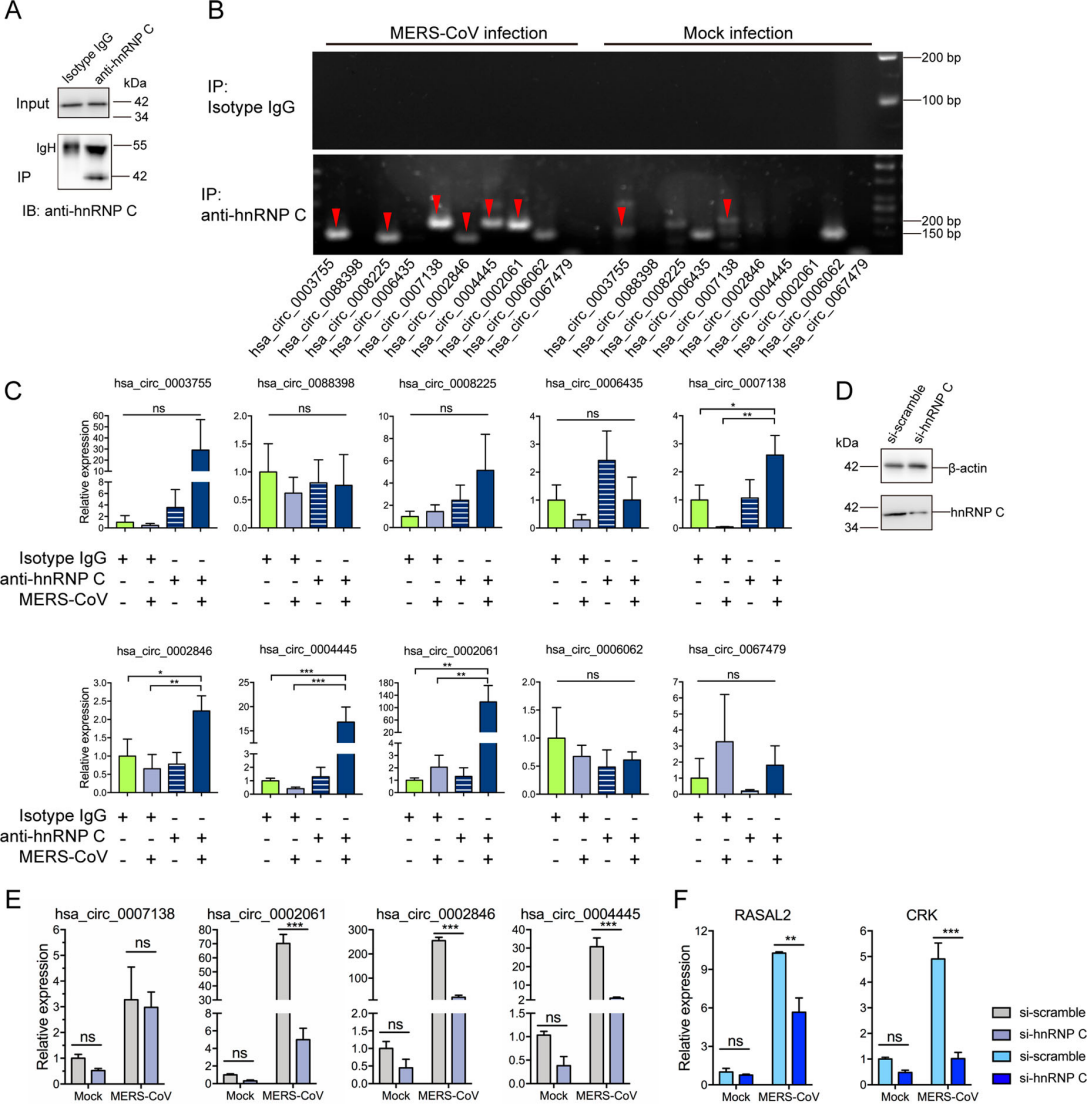
hnRNP C regulates the expression of MERS-CoV-perturbed circRNA and cognate mRNAs via direct binding. (A) Representative Western blot examining hnRNP C protein in input and immunoprecipitation (IP) samples for RNA immunoprecipitation (RIP) assay. Isotype antibody was used as a negative control to assess the binding specificity between hnRNP C and each circRNA. IB: immunoblotted. IgH: IgG heavy chain. (B) Representative gel electrophoresis validation of circRNAs immunoprecipitated using anti-hnRNP C and isotype-matched IgG antibody in Calu-3 cells with or without MERS-CoV infection. The red arrowheads indicated the immunoprecipitated circRNA. (C) The relative amount of circRNAs enriched from RIP. The quantification was made from the RT-qPCR results and by normalizing the expression values of circRNAs in IP samples with the expression values in input control samples. (D) Western blot demonstrated the efficient knockdown of hnRNP C in Calu-3 cells. (E, F) Expression changes of representative circRNAs (E) and cognate mRNAs (F) upon hnRNP C knockdown. Data from (C) represented mean ± standard deviation, *n* = 3. *P-value < 0.05; **P-value < 0.01; ***P-value < 0.001, one-way ANOVA. Data from (E) and (F) represented mean ± standard deviation, *n* = 3. *P-value < 0.05; **P-value < 0.01; ***P-value < 0.001, two-way ANOVA. Abbreviation: ns, not significant.

Circularized exons are more likely to contain complementary Alu sequences than non-circularized exons [36]. hnRNP C has been shown to bind and obscure Alus on pre-mRNAs and protect against Alu exonization to regulate circRNA biogenesis [9, 35]. The biogenesis of circRNAs is diversified in different cells and may be impacted by various stimuli [30]. Thus, whether hnRNP C functions preferably on the virus-regulated circRNAs compared to other circRNAs is unclear. As hnRNP C demonstrated the most extensive interaction with MERS-CoV-triggered circRNAs, we prioritized it as a key regulator of the expression of circRNAs and their cognate mRNAs during MERS-CoV infection. To this end, we first assessed whether hnRNP C could bind to the 10 identified circRNAs through RIP assays. Mock-infected and MERS-CoV-infected Calu-3 cells were lysed and immunoprecipitated with anti-hnRNP C antibody or isotype-matched IgG antibody (Figure 3A and 3B). The amount of immunoprecipitated circRNA was quantified by RT-qPCR (Figure 3C) and normalized with the value in input samples (Supplementary Figure 3). Among the 10 tested circRNAs, hsa_circ_0007138, hsa_circ_0002846, hsa_circ_0004445, hsa_circ_0002061, and hsa_circ_0006435 were shown to specifically bind to hnRNP C (Figure 3B and 3C). To further define hnRNP C's modulatory role on these circRNAs, we knocked down hnRNP C (Figure 3D) and detected the expression of its target circRNAs and mRNAs by RT-qPCR. Based on the quantitation of RIP by RT-qPCR (Figure 3C), we prioritized 3 circRNAs that significantly bound to hnRNP C and were significantly decreased when hnRNP C was knocked down for further analysis. These 3 circRNAs were hsa_circ_0002846 and hsa_circ_0002061 [both originating from RAS protein activator like 2 gene (RASAL2)], and hsa_circ_0004445 (originating from CRK) (Figure 3E). In accordance with our hypothesis, the knockdown of hnRNP C restricted the expression of RASAL2 and CRK as well (Figure 3F). Taken together, these results validated the role of hnRNP C in the expression of circRNAs and their cognate mRNAs during MERS-CoV infection. To test the specificity of the regulations of hnRNP C, we knocked down the individual splicing factors identified in our RBP-circRNA network (Figure 2C) and examined the expression of each circRNAs (Supplementary Figure 4). Our RT-qPCR results showed that the expression of hsa_circ_0002846, hsa_circ_0002061, and hsa_circ_0004445, which were targeted by hnRNP C, did not significantly change after knocking down SNRNP70 (Supplementary Figure 4).

### Suppression of hnRNP C more potently inhibits MERS-CoV replication than depletion of specific circRNAs

**Figure 4.**
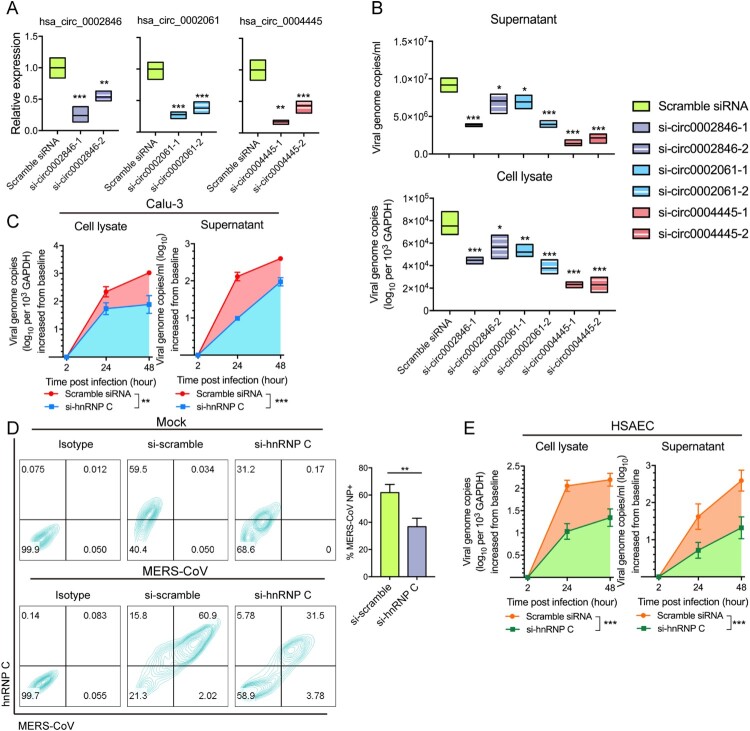
Depletion of hnRNP C and its target circRNAs restrict MERS-CoV propagation. (A) RT-qPCR examining the knockdown effect of siRNA candidates targeting hsa_circ_0002846, hsa_circ_0002061 and hsa_circ_0004445, respectively. Expression of GAPDH was used to normalize the quantification results. (B) Viral load detection of cells transfected with scramble control siRNAs, siRNAs targeting hsa_circ_0002846, hsa_circ_0002061 and hsa_circ_0004445, respectively at 24 hours post MERS-CoV infection (MOI = 0.1). (C) Multi-cycle MERS-CoV growth assay in the presence or absence of hnRNP C siRNA in Calu-3 cells. After inoculated with MERS-CoV (MOI = 0.1), culture supernatant and cell lysate were harvested at 2, 24, and 48 hpi to determine the viral load with RT-qPCR. Area under the curve analysis was conducted to reveal the total amount of viral genome copy increase over 2–48 hours period. Statistical significance was determined with Student's test. *P-value < 0.05; **P-value < 0.01; ***P-value < 0.001. (D) Flow cytometry examining MERS-CoV-NP-positive cells with or without hnRNP C depletion. Representative expression of endogenous hnRNP C and MERS-CoV-NP were plotted in left panel. Triplicated experiments were conducted for the quantification of the MERS-CoV-NP positive rate. (E) Multi-cycle MERS-CoV growth assay in the presence or absence of hnRNP C siRNA in primary human small airway epithelial cells (HSAECs). Culture supernatant and cell lysate were harvested at 2, 24, and 48 hours post MERS-CoV infection (MOI  = 0.1) and subjected to RNA extraction and RT-qPCR measurement. Area under the curve analysis was conducted same as described above. All data represented mean ± standard deviation, n = 3. Statistical significance analyses for (A), (B), (D), and (E) were determined with one-way ANOVA. *P-value < 0.05; **P-value < 0.01; ***P-value < 0.001.

Recent studies have uncovered the role of circRNAs as host pathogenic factors utilized by multiple viruses [10, 11, 37]. However, manipulation of host circRNAs does not consistently provide satisfactory effects on restricting virus replication, which may be ascribed to the relative low expression level of circRNAs [37]. Since we have pinpointed hnRNP C as an upstream regulator of multiple proviral circRNAs and mRNAs, we asked whether modulating this central splicing factor may generate more prominent impact on virus propagation. To validate our hypothesis, we compared the viral load inhibitory effects between siRNA-silencing of hnRNP C and silencing of its individual target circRNAs, namely hsa_circ_0002846, hsa_circ_0002061, and hsa_circ_0004445. We designed a set of siRNAs targeting the back-splicing site of hsa_circ_0002846, hsa_circ_0002061, and hsa_circ_0004445 through CircInteractome (https://circinteractome.nia.nih.gov/). Then, we synthesized and transfected them into Calu-3 cells (Figure 4A) and found that the depletion of these circRNAs could reduce MERS-CoV replication (Figure 4B). Among the three circRNAs, knockdown of hsa_circ_0004445 demonstrated the most potent inhibitory effect on MERS-CoV replication in both cell lysate and culture supernatant by about 50-75% (Figure 4B). Knockdown of these circRNAs did not influence the expression of hnRNP C with or without influencing the expression of its cognate mRNA (Supplementary Figure 5). While off-target effect is a possibility, this has not been well described for circRNA knockdown in the literature. In parallel, depletion of hnRNP C resulted in over 50% inhibition of MERS-CoV replication in both cell lysate and supernatant samples collected at 24 and 48 hpi (*P* < 0.05 to *P* < 0.001). The inhibition of viral load was over 1 log in supernatant samples collected at 24 hpi (Figure 4C). Moreover, flow cytometry showed that the percentage of cells expressing MERS-CoV NP decreased from 61.86% (scrambled siRNA-transfected cells) to 36.76% (hnRNP C siRNA-transfected cells) at 24 hpi after hnRNP C knockdown (Figure 4D). MERS-CoV infection predominantly presents with respiratory tract symptoms [38–40]. For further clinical correlation, we knocked down hnRNP C in the more physiologically relevant primary cells HSAECs. As shown in Figure 4E, hnRNP C knockdown in HSAECs resulted in significant MERS-CoV viral load reduction in both cell lysate and supernatant (*P* < 0.001) by the area-under-the-curve calculation. hnRNPs could directly interact with viral RNAs, structural and non-structural proteins [41, 42]. Previous reports have shown specific *in vitro* binding between human hnRNP A1 and SARS-CoV NP [43], as well as the binding between hnRNP I (polypyrimidine tract-binding protein) and positive-sense RNAs of transmissible gastroenteritis coronavirus [44]. In the present study, hnRNP C showed distinct cellular localization with MERS-CoV NP, which indicated that lack of direct interactions between hnRNP C and MERS-CoV NP (Supplementary Figure 6). Together, our results underscored the essential role of hnRNP C in MERS-CoV replication, especially in physiologically relevant cells. Inhibition of hnRNP C demonstrated prominent effects on restricting MERS-CoV propagation, likely due to its central role in the regulation of multiple proviral circRNAs and mRNAs.

### hnRNP C is a common proviral factor for human-pathogenic coronaviruses including MERS-CoV, SARS-CoV-2, and SARS-CoV

**Figure 5.**
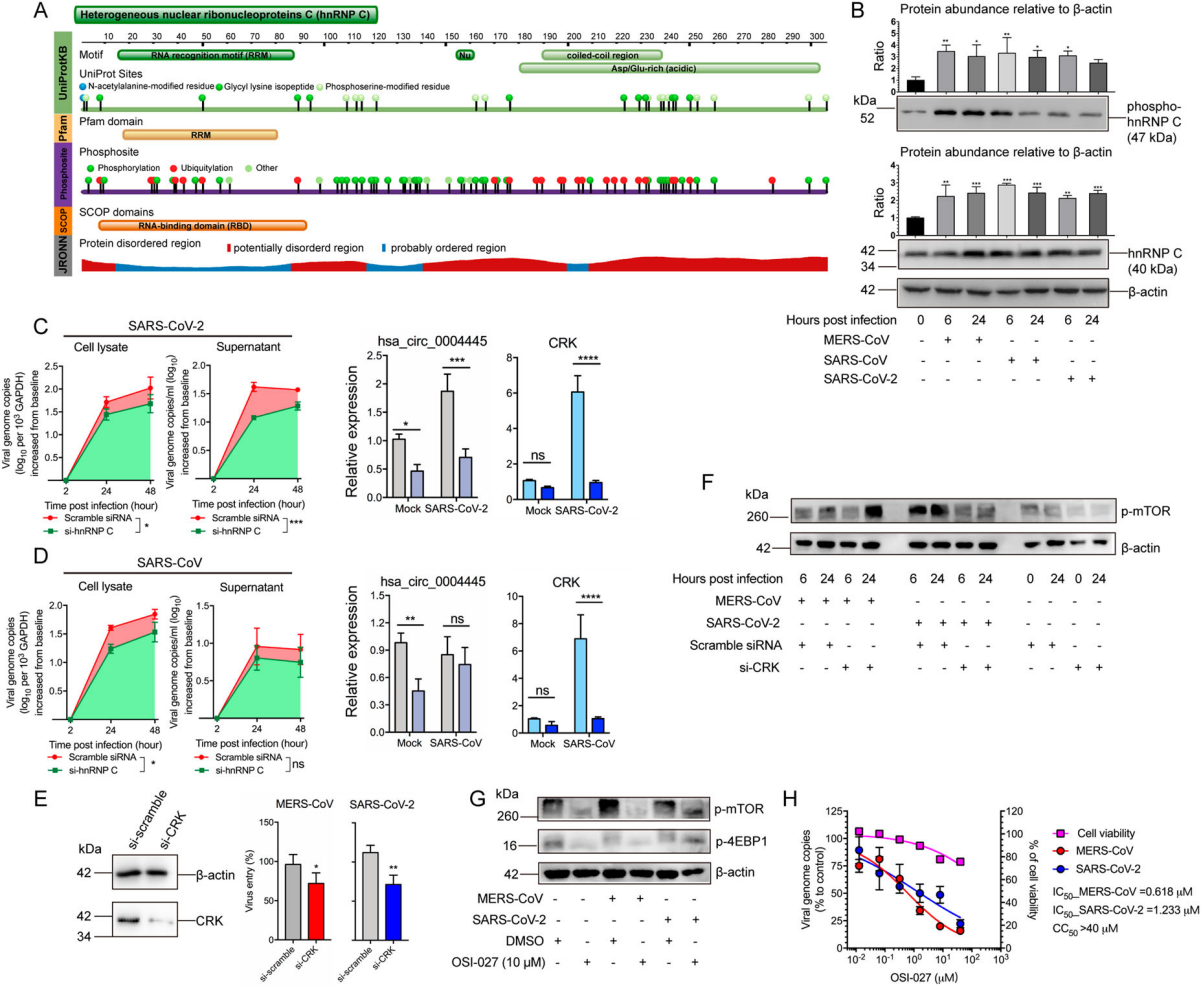
The modulation of hnRNP C in human-pathogenic coronavirus infections. (A) Demonstration of the representative structural features of hnRNP C by UniProtKB, Pfam, Phohsphosite, SCOP, and JRONN database. Protein feature view was adopted from the Protein Data Bank (PDB; http://www.rcsb.org/pdb/). (B) hnRNP C was upregulated and underwent phosphorylation modification upon MERS-CoV, SARS-CoV, and SARS-CoV-2 infection. The expression intensities of hnRNP C and phosphorylated hnRNP C in each sample were quantified in western blot images by ImageJ. (C) hnRNP C knockdown suppressed SARS-CoV-2 genomic copies intracellularly and extracellularly, and reduced the expression of hsa_circ_0004445 and CRK. (D) hnRNP C knockdown suppressed SARS-CoV genomic copies intracellularly and reduced the expression of CRK. Scramble siRNA-transfected cells were served as negative controls. Area under the curve analysis was conducted in the same way as described above. (E) Ablation of CRK restricted MERS-CoV and SARS-CoV-2 entry. After performing siRNA-mediated gene silencing for CRK for consecutive two days, Calu-3 cells were challenged with MERS-CoV or SARS-CoV-2 at 3.0 MOI respectively. Samples were harvested at 4 hpi to examine the viral loads. (F) Expression changes of phosphorylated mTOR (p-mTOR) upon hnRNP C knockdown in MERS-CoV-, SARS-CoV-2-, and mock-infected groups, respectively. (G) Expression of p-mTOR and phosphorylated 4EBP1 (p-4EBP1) decreased upon OSI-027 treatment. (H) Calu-3 cells were infected with MERS-CoV or SARS-CoV-2 and treated with various concentrations of OSI-027 (40, 8, 1.6, 0.32, 0.064, 0.0128 μM, and mock-treated). The IC_50_ for each virus was calculated based on viral load changes at 24 hpi. CC_50_ was determined in the absence of virus infection at 24 hour post drug treatment. Data represented mean ± standard deviation, *n* = 3. one-way ANOVA, *P-value < 0.05; **P-value < 0.01; ***P-value < 0.001.

Typically identified as an interactor of viral RNAs with an altered localization in infected cells, hnRNP C modulated different steps of infection initiated by multiple virus species [45–47]. While hnRNP C is known as a splicing factor, its function during coronavirus infection remains largely unknown. hnRNP C was reported to be phosphorylated in HeLa cells by casein kinase 2 and in human endothelial cells in response to hydrogen peroxide exposure [48, 49], endowed with multiple phosphorylation sites as well as a large proportion of intrinsically disordered domains (IDDs) (Figure 5A). Post-translational modifications including protein phosphorylation substantially determined the capacity of splicing factors such as HNRNPM to interact with RNAs or proteins [50]. IDDs would advantageously provide protein–protein and protein–RNA interaction interfaces, thus are usually enriched in RNA-interactive proteins [51]. These unique structural characteristics strongly emphasize the intensive association within the phosphorylation of hnRNP C and the biogenesis of circRNAs during virus infection. To comprehensively profile the involvement of hnRNP C in coronavirus infection as a key splicing regulator, we simultaneously examined the expression and phosphorylation of hnRNP C in MERS-CoV, SARS-CoV, and SARS-CoV-2 infections. As shown in Figure 5B, both the phosphorylated form and total amount of hnRNP C increased upon MERS-CoV, SARS-CoV, and SARS-CoV-2 infection at two different time points (6 and 24 hpi). This suggested that hnRNP C might be a potential broad-spectrum anti-coronaviral target. Hence, we investigated the virus replication kinetics of SARS-CoV and SARS-CoV-2 in Calu-3 cells transfected with hnRNP C or scrambled siRNA. As shown in Figure 5C, the viral load in si-hnRNP C transfected Calu-3 cells reduced intracellularly (*P* < 0.05) and extracellularly (*P* < 0.001) relative to scrambled siRNA-transfected cells during SARS-CoV-2 infection. This inhibition might have resulted from the similar alterations of MERS-CoV-perturbed proviral circRNAs and cognate mRNAs, as we observed diminished hsa_circ_0004445 and its cognate gene CRK after hnRNP C knockdown during SARS-CoV-2 infection, similarly to MERS-CoV infection (Figure 5C). Similar to SARS-CoV-2, hnRNP C knockdown attenuated SARS-CoV replication intracellularly (*P* < 0.05) but did not significantly suppress the expression of hsa_circ_0004445 during SARS-CoV infection (Figure 5D), which might suggest differences in the function of hnRNP C in MERS-CoV, SARS-CoV-2, and SARS-CoV infections. Taken together, our findings indicated that while modulations of hnRNP C exhibited proviral roles in different human-pathogenic coronaviruses, the downstream virus-perturbed circRNAs and cognate mRNAs may vary among different viruses.

hnRNP C's overall inhibitory effects on virus replication may largely depend on how its target circRNAs and cognate mRNAs individually function. As the functions of most circRNAs remain unknown, dissection of the functions of their co-expressed mRNAs may help to reveal the effects of hnRNP C. In the present study, hnRNP C knockdown significantly decreased the expression of hsa_circ_0004445 and its cognate mRNA CRK during MERS-CoV and SARS-CoV-2 infections. We therefore further investigated the biological relevance of CRK depletion in MERS-CoV and SARS-CoV-2 infections and the role of CRK as a potential broad-spectrum anti-coronaviral target. As the Abl2 inhibitor imatinib was reported to impact MERS-CoV replication within 4 hpi [52], we postulated that CRK depletion might affect virus replication during the early stage of virus infection as well. As shown in Figure 5E, CRK knockdown with siRNAs resulted in significantly reduced entry of both MERS-CoV by about 28.1% (*P* < 0.05) and SARS-CoV-2 by about 28.6% (*P* < 0.01). We observed that CRK depletion negatively regulated the phosphorylation of mTOR during MERS-CoV and SARS-CoV-2 infections (Figure 5F).

To further validate whether the inhibition of the CRK-mTOR pathway accounted for the suppressed virus propagation, we evaluated the antiviral effect of a specific mTOR inhibitor, OSI-027, which is under clinical trial for patients with advanced solid malignancies [53] against MERS-CoV and SARS-CoV-2 in Calu-3 cells. To test the specificity of OSI-027 in Calu-3 cells, we examined the expression of phosphorylated mTOR (p-mTOR) and its target protein eIF4E-binding protein 1 (4EBP1). Our results showed that the activation of mTOR and 4EBP1 were suppressed upon OSI-027 treatment as evidenced by the decreased expression of p-mTOR and p-4EBP1 (Figure 5G). The CC_50_ in Calu-3 cells was over 40 μM. In line with our hypothesis, OSI-027 caused dose-dependent inhibition of the replication of both MERS-CoV and SARS-CoV-2 at non-cytotoxic concentrations, with IC_50_ of 0.618 and 1.233 μM for MERS-CoV and SARS-CoV-2, respectively (Figure 5H).

## Discussion

In the past two decades, three novel coronaviruses, namely, SARS-CoV, MERS-CoV, and SARS-CoV-2, have emerged to cause epidemics or pandemic with significant public health and socioeconomic impact [40, 54, 55]. Studies on virus-host interactions are essential for deciphering the pathogenesis and developing treatment strategies for coronavirus infections. Recent evidences have highlighted the role of circRNAs as important regulators of gene expression through acting as miRNA sponges or interacting with RBPs, allowing them to be exploited by viruses to enhance viral replication [2–4]. As fine-tuners of circRNA biogenesis, RBPs functioning as splicing factors is likely to be also involved in virus infection as proviral factors. Our previous study revealed that the percentage of circRNAs that were co-expressed with their cognate mRNA was 8.1% before MERS-CoV infection, while around 61.3% of DE circRNAs co-expressed with their cognate mRNAs after MERS-CoV infection [12]. As both circRNAs and mRNAs are splicing products, we hypothesized that the expression of host circRNAs and their cognate mRNAs may be mediated by splicing factors in MERS-CoV infection. In this study, we first conducted a comprehensive *in silico* analysis to investigate the interactions between MERS-CoV-perturbed splicing factors and representative DE circRNAs-mRNA pairs. Combining the results from correlation analysis and binding motif predictions, we identified seven splicing factors and validated hnRNP C as a key regulator of MERS-CoV-perturbed circRNAs and cognate mRNAs.

hnRNP C has been reported to facilitate dengue virus, Japanese encephalitis virus, and poliovirus infections as a proviral factor through interacting with viral RNAs or non-structural proteins [45–47]. The data in this study revealed a novel role of hnRNP C in the modulation of host circRNAs and their cognate mRNAs in coronavirus infections. Our RIP results confirmed the direct binding between hnRNP C and five representative MERS-CoV-perturbed circRNAs (hsa_circ_0002846, hsa_circ_0007138, hsa_circ_0002061, hsa_circ_0004445, and hsa_circ_0006435). Depletion of hnRNP C by siRNA significantly reduced the expression of hsa_circ_0002846, hsa_circ_0002061, and hsa_circ_0004445, and their cognate mRNAs (Figure 3). Physical binding is a feature in almost all the interactions between splicing factors and circRNAs. For example, muscleblind regulates the circularization of its own pre-mRNA through direct binding to the flanking intronic sequences giving rise to circRNAs [5]. QKI regulates the formation of circRNAs associated with human epithelial mesenchymal transition via QKI-binding motifs [6]. Multiple HNRNPs and SR proteins interact with intronic repeats combinatorially as a pre-mRNA splicing machinery on the circularization of the Drosophila laccase2 gene [7]. FUS regulates circRNA biogenesis by binding to the introns flanking the back-splicing position [8]. hnRNP C was reported to regulate circRNA biogenesis via direct binding to the 3′ back-splice sites of pre-mRNAs in human cancer cells under hypoxic stress [9]. Our findings demonstrated that hnRNP C modulated the expression of MERS-CoV-perturbed circRNAs and cognate mRNAs through direct physical binding.

In addition to identifying the role of hnRNP C in regulating the expression of circRNAs and cognate mRNAs in MERS-CoV infection, we also showed that hnRNP C regulated the replication of MERS-CoV. siRNA knockdown of hnRNP C led to significantly reduced expression of CRK and the phosphorylation of mTOR. Members of the CRK family are typically known as adaptor proteins that function by forming various signal transduction protein complexes with receptor tyrosine kinases utilizing their Src homology 2 (SH2) and Src homology 3 (SH3) domains upon different stimuli [56]. Our present findings helped to explain the previously reported *in vitro* anti-coronaviral effects of the mTOR inhibitor rapamycin and the PI3 K/Akt/mTOR pathway inhibitor wortmannin [57, 58]. Corroborating with these reports, we showed that the mTOR inhibitor OSI-027, an orally available anti-cancer drug currently being evaluated in Phase I clinical trial for advanced solid malignancies [53], exhibited significant antiviral activity against both MERS-CoV and SARS-CoV-2.

Collectively, our study identified a novel role of the RBP hnRNP C in the regulation of MERS-CoV-perturbed circRNAs. The intervention of the downstream signalling pathway of hnRNP C by the use of the mTOR inhibitor OSI-027 led to significantly reduced coronavirus replication. Further *in vivo* evaluation of the effect of hnRNP C inhibition or inhibitors targeting the CRK-mTOR signaling pathway should be considered for MERS-CoV and other coronavirus infections.

## Supplementary Material

Supplemental Material
