# Supplementary material for: hnRNP C modulates MERS-CoV and SARS-CoV-2 replication by governing the expression of a subset of circRNAs and cognitive mRNAs
Source: Emerg Microbes Infect. 2022 Feb 10;11(1):519–31. doi: 10.1080/22221751.2022.2032372 (PMC8843244; doi:10.1080/22221751.2022.2032372)
Supplement: Supplemental Material [file TEMI_A_2032372_SM2191.zip › Suppl files/Supplementary_Materials_2022_01_11_cleancopy.docx]

**SUPPLEMENTARY MATERIALS**

**Supplementary Figure 1.** Dot plot demonstrating the correlations between circRNAs identified in mock-infected Calu3 cells and their cognitively expressed mRNAs (left), as well as the DE circRNAs and their corresponding mRNAs in MERS-CoV infected cells (right). Dots represent circRNAs and were colored according to their correlations with respective mRNAs at 24 hpi.

**Supplementary Figure 2.** RT-qPCR validation of the representative MERS-CoV perturbed circRNAs (Supplementary Figure 2A) and mRNAs (Supplementary Figure 2B). Calu-3 cells were either mock-infected or infected with MERS-CoV (MOI = 0.1). At 24 hpi, cell lysates were harvested for the detection of each circRNA and mRNA. Linear RNA of GAPDH was used as an internal reference to normalize the RNA expression.

**Supplementary Figure 3.** circRNA expression in input samples. The abundance of each circRNA in input samples of RIP was quantified by qRT-PCR which was used to normalize the results of immunoprecipitated circRNAs.

**Supplementary Figure 4.** circRNA expression profile of knockdown of splicing factors. Calu-3 cells were transfected with scramble siRNAs or siRNAs targeting TRA2B, HNRNPM, SRSF2, SNRNP70, SNRPA, U2AF2, respectively to examine the specificity of the regulations of each splicing factor. Linear RNA of GAPDH was used as an internal reference to normalize the RNA expression. Data represented mean ± standard deviation, n = 3, two-way ANOVA, *P < 0.05; **P < 0.01; ***P < 0.001.

**Supplementary Figure 5.** Calu-3 cells transfected with scramble siRNAs or siRNAs targeting circ0002846, circ0002061, or circ0004445, were used to evaluate the impact of circRNA knockdown on the expression of (A) their respective cognate mRNA by RT-qPCR and (B) hnRNP C by Western blot. Data represented mean ± standard deviation. For the evaluation of RASAL2, n = 3, one-way ANOVA, **P < 0.01; for the evaluation of CRK, n = 3, Student’s t-test, ***P < 0.001.

**Supplementary Figure 6.** hnRNP C did not co-localize with MERS-CoV nucleocapsid protein (NP) intracellularly. Calu-3 cells were infected by MERS-CoV (MOI = 0.1) and fixed at 24 hpi for immunofluorescence staining.

**Supplementary Table 1.** Primers and customized siRNA sequences in the study.
