# Supplementary material for: hnRNP C modulates MERS-CoV and SARS-CoV-2 replication by governing the expression of a subset of circRNAs and cognitive mRNAs
Source: Emerg Microbes Infect. 2022 Feb 10;11(1):519–31. doi: 10.1080/22221751.2022.2032372 (PMC8843244; doi:10.1080/22221751.2022.2032372)
Supplement: Supplemental Material [file TEMI_A_2032372_SM2191.zip › Suppl files/Supplementary_Table_1_editable.docx]

Supplementary Table 1 siRNAs

| **Name** | **sense** | **antisense** |
| --- | --- | --- |
| si-circ0004445-1 | GCC UUG GCU UUG GAG GGG UGA | UCA CCC CUC CAA AGC CAA GGC |
| si-circ0004445-2 | GCU UUG GAG GGG UGA GCC CCU | AGG GGC UCA CCC CUC CAA AGC |
| si-circ0002846-1 | GCC ACU AAA CUG GAU GUG AAA | UUU CAC AUC CAG UUU AGU GGC |
| si-circ0002846-2 | GCG CCA CUA AAC UGG AUG UGA | UCA CAU CCA GUU UAG UGG CGC |
| si-circ0002061-1 | GGC ATC TGC TTC TGA TCA TCT | AGA UGA UCA GAA GCA GAU GCC |
| si-circ0002061-2 | GCU UCU GAU CAU CUA UCU AAA | UUU AGA UAG AUG AUC AGA AGC |

Primers

| **Name** | **Forward primer** | **Reverse primer** |
| --- | --- | --- |
| GAPDH | ATTCCACCCATGGCAAATTC | CGCTCCTGGAAGATGGTGAT |
| hsa_circ_0003755 | ATGTCAGTGTGGACTCCGTG | TAGCTGGGAGGGTGGTCTTA |
| hsa_circ_0067479 | TGGTGGACAGTTTATGGGAGAG | ACTGTCATTTAGGAAGGCATCACT |
| hsa_circ_0002846 | AACTGTCAGTGTCCCTTCCG | TGTCTCGGTGTAGGGTGACT |
| hsa_circ_0006062 | ACTCATGGAGAGGGAGATGTGA | ATCATCCTGATGGGGTGGGG |
| hsa_circ_0007138 | ACTCCTTGGGAATTTAGCGTTCT | TGCAGTGAGTGTCGTTCTCC |
| hsa_circ_0002061 | GGAATACCTGAGGCTGGGTAA | GGTGAGAGATGGGTGAAGGA |
| hsa_circ_0088398 | TGTCAACTTCGCACTTCTCCA | TCTGTCCATTGAGCTGCCTG |
| hsa_circ_0004351 | AGGAACTTCTGGATCCTGCG | TCTGCAGGTGTGCCTCATTT |
| hsa_circ_0004445 | AACACTCCGCTCCCTAACCT | TCCAGTAAAGCAGGCAATGA |
| hsa_circ_0008225 | AGAGACAGCAGTAGTCCCTGG | ATCTCAATGGCTGCCCAAAGA |
| hsa_circ_0006435 | TCGGTAGCACCAATGAAAAGA | GACACCTGGGAGCTTGCTAT |
| FAM208B | AAAGGTGAACGCAAAGCCAT | CCCTCCCCACTGTTCTCAAT |
| STAG1 | AGGATTGCAGCTCCGTTGAA | AGTGCGATGTCCCTGTCTTG |
| RASAL2 | TCCAGGGATGCAGGTTTCAT | ATGGGTCAGCTTCGAAGTGA |
| DYRK1A | ACTTAACAGGGAGGGACAGC | TAAGGAGGCTTGTGAGTGGG |
| PTPRK | GCAGCAGCAACAGAAGAACC | GCGAGGTACGTCTAGAAGGC |
| RC3H2 | GCTCGCTTGAACCAGAGTGC | CTGAGGGTTCTGGTGCTGTA |
| ZNF398 | CGGTCGAAGACGAGATGAGG | CCTCTCAGTCCGGGTTTCTG |
| CRK | GAACAGGCAGCGCTACTCA | CCTTTGATTTCTTGAGGGGCA |
| ZMYND11 | CAACGTAGCAATCTCCAGCC | TTTGCGAAATACTAGCGGGC |
| UGCG | TGGATTATCCCAAATATGAAGTGCT | TCGGTCAGCTATCGCTTTGG |
