# Supplementary figures and images for: hnRNP C modulates MERS-CoV and SARS-CoV-2 replication by governing the expression of a subset of circRNAs and cognitive mRNAs
Source: Emerg Microbes Infect. 2022 Feb 10;11(1):519–31. doi: 10.1080/22221751.2022.2032372 (PMC8843244; doi:10.1080/22221751.2022.2032372)

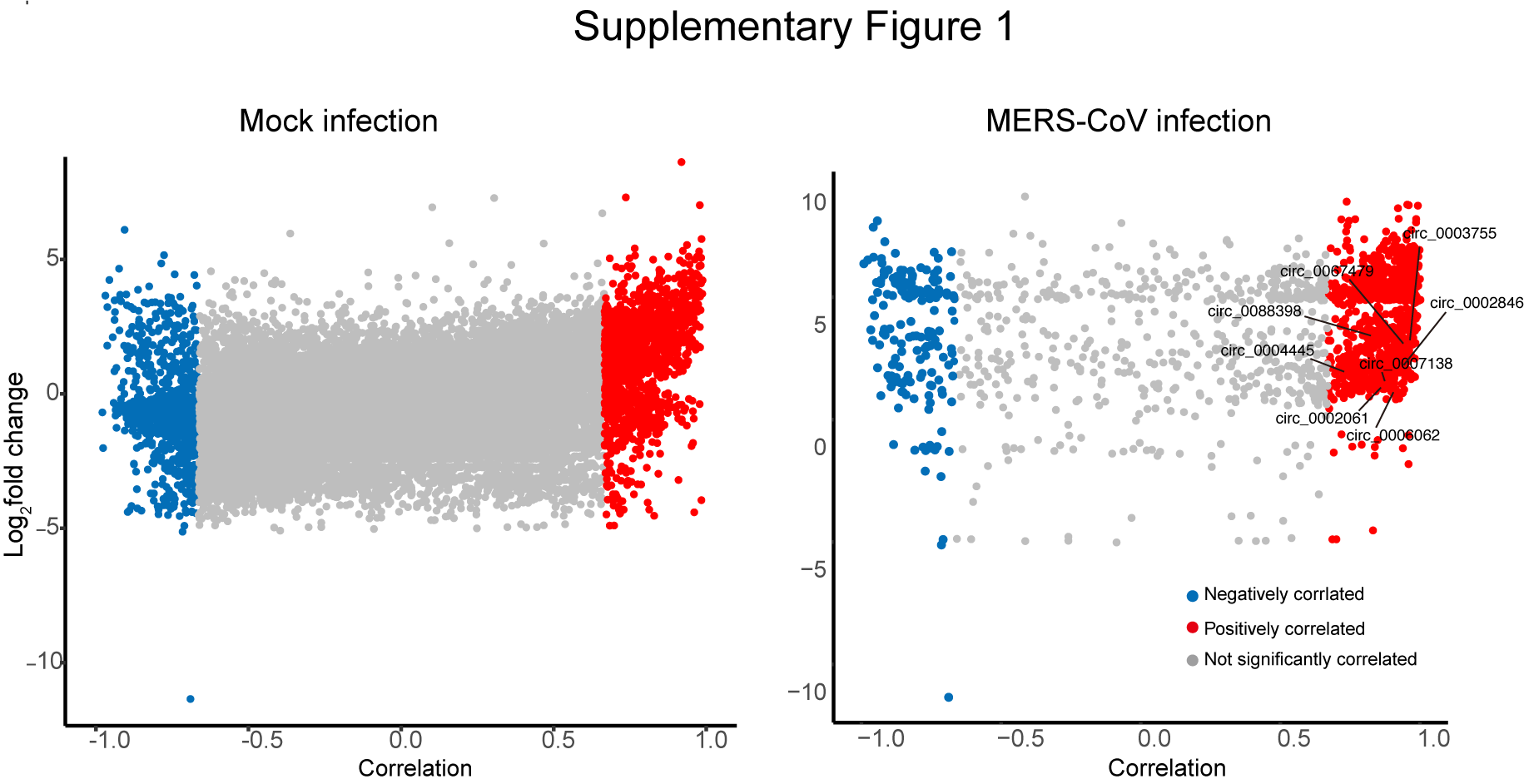

Supplement: Supplemental Material [file TEMI_A_2032372_SM2191.zip › Suppl files/Supplementary Figure 1.png]

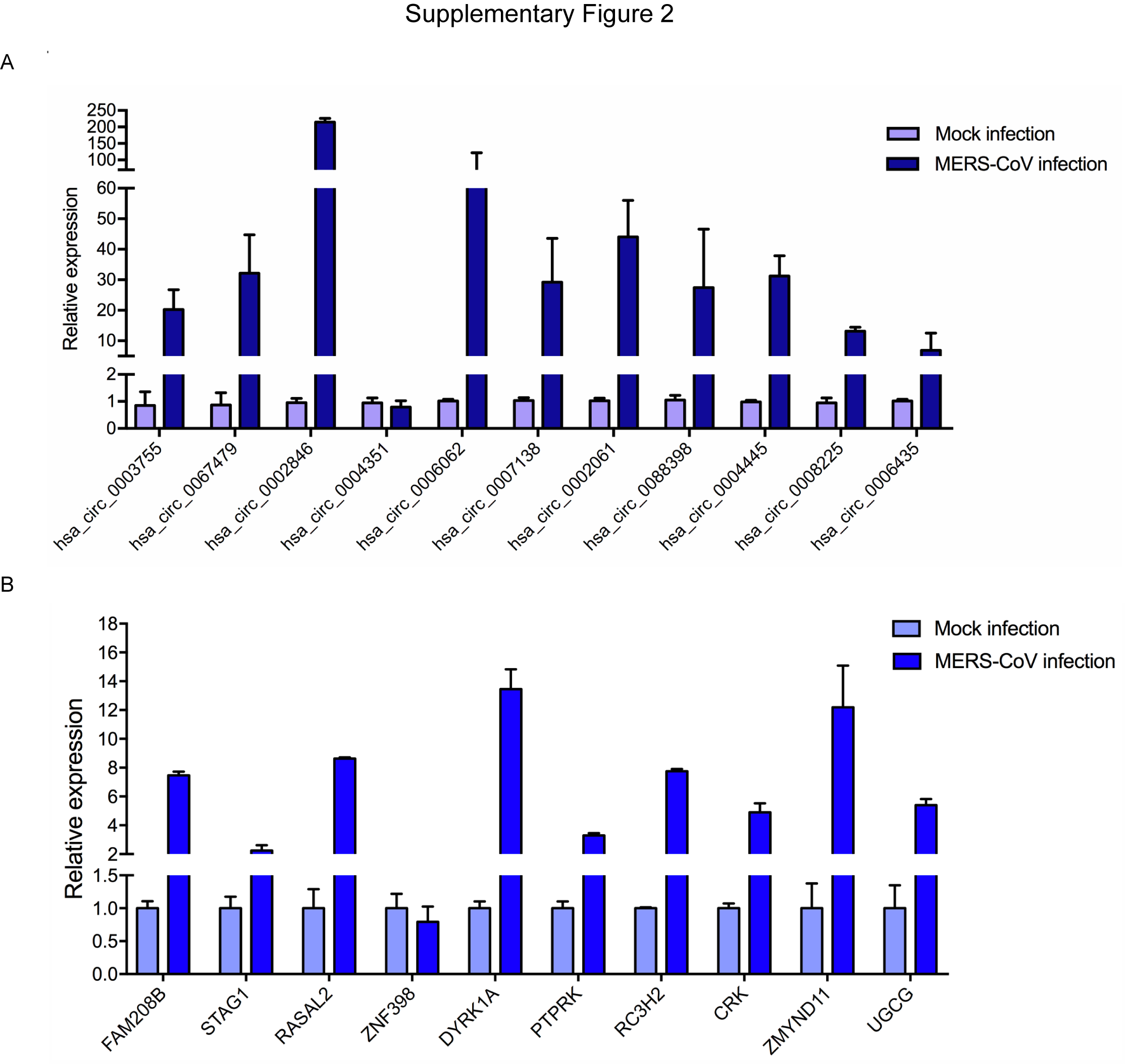

Supplement: Supplemental Material [file TEMI_A_2032372_SM2191.zip › Suppl files/Supplementary Figure 2.png]

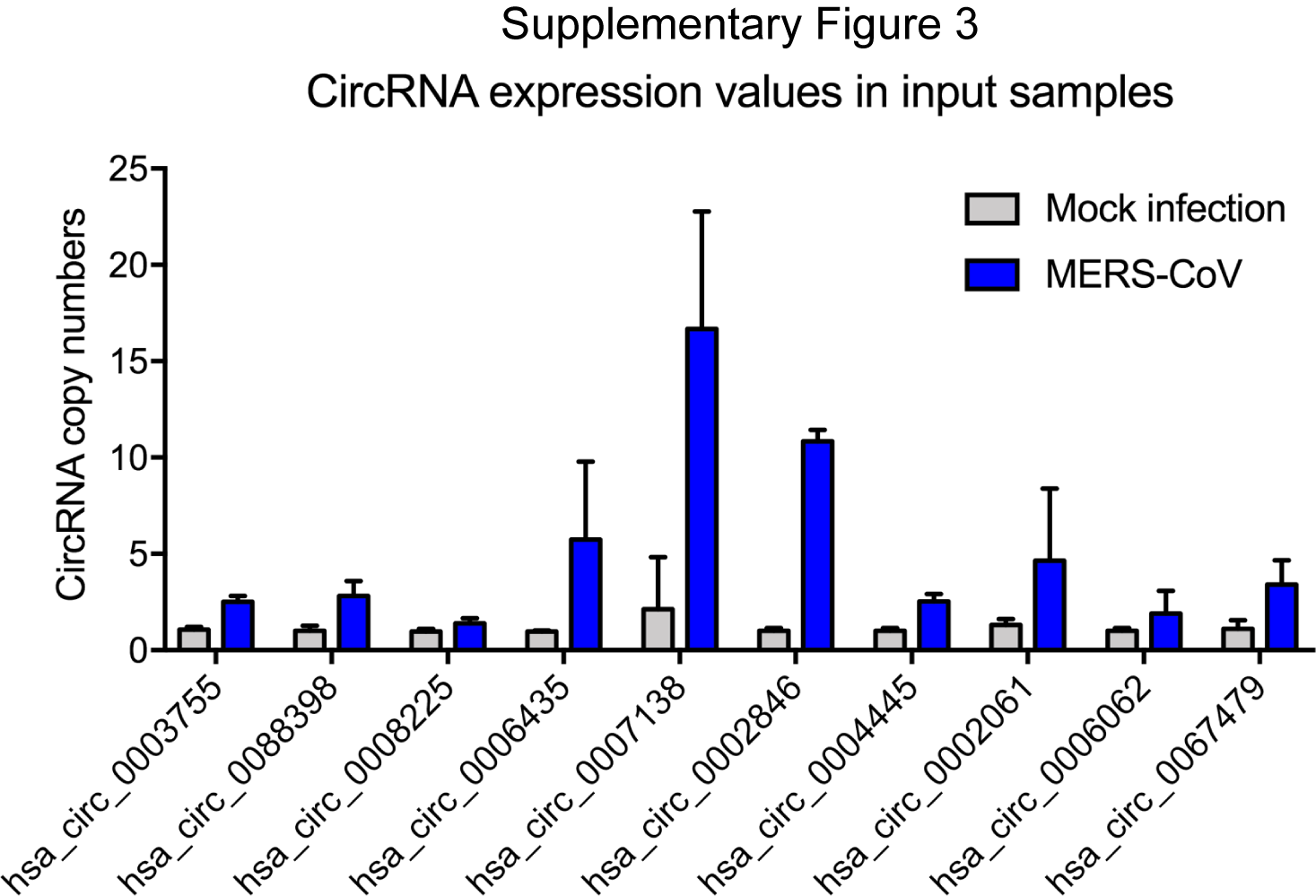

Supplement: Supplemental Material [file TEMI_A_2032372_SM2191.zip › Suppl files/Supplementary Figure 3.png]

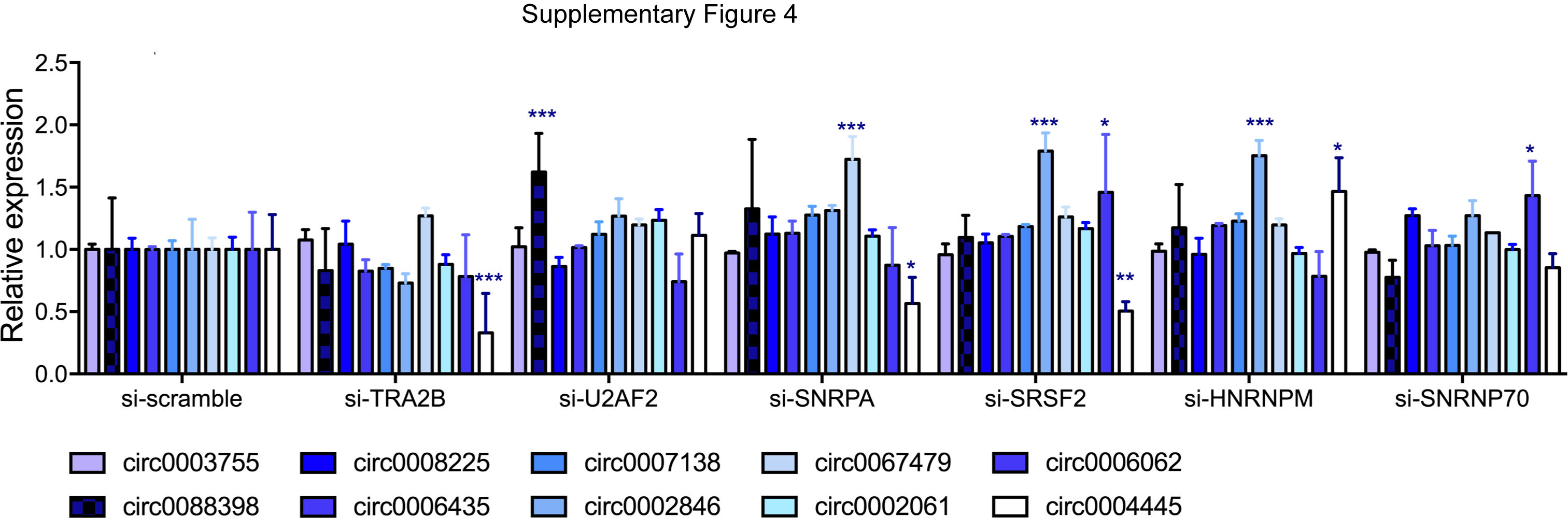

Supplement: Supplemental Material [file TEMI_A_2032372_SM2191.zip › Suppl files/Supplementary Figure 4.png]

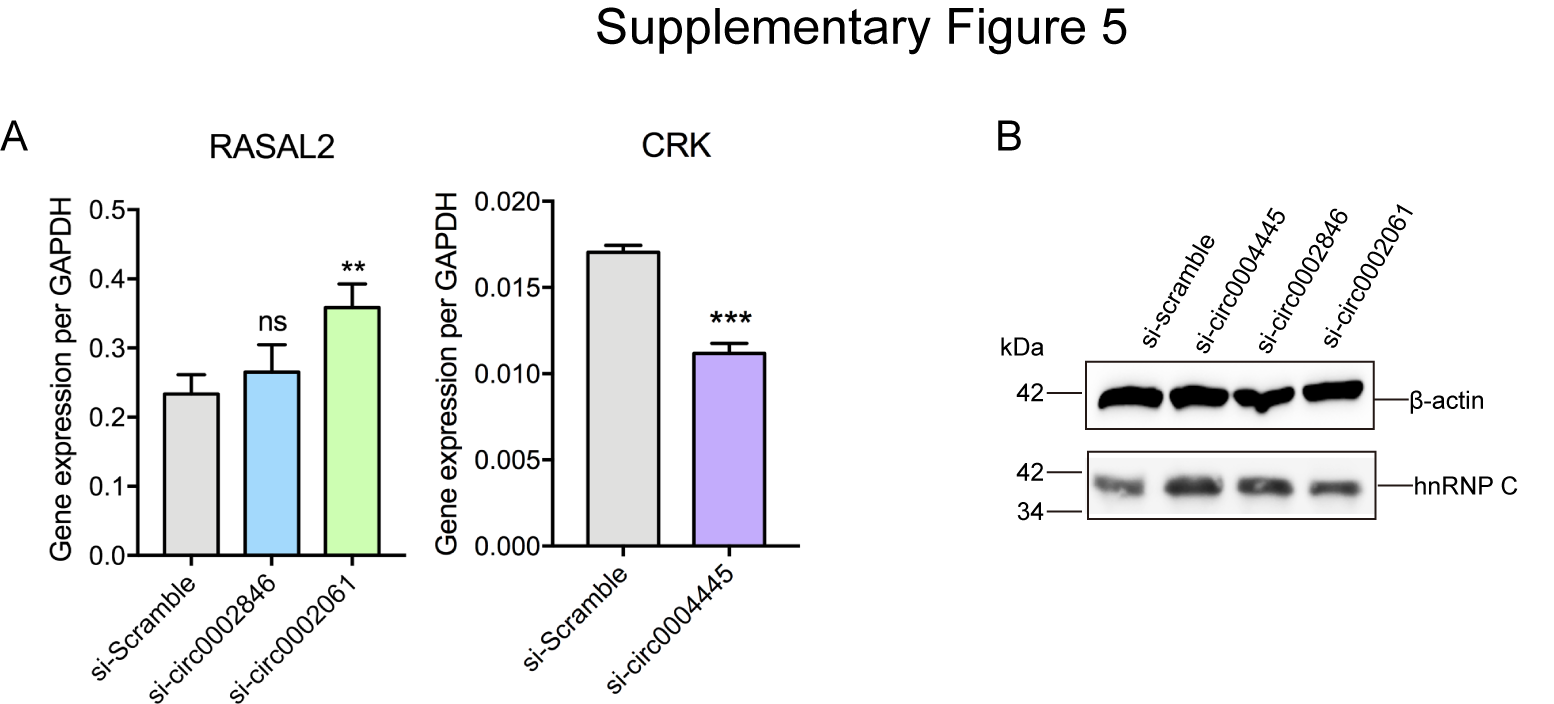

Supplement: Supplemental Material [file TEMI_A_2032372_SM2191.zip › Suppl files/Supplementary Figure 5.png]

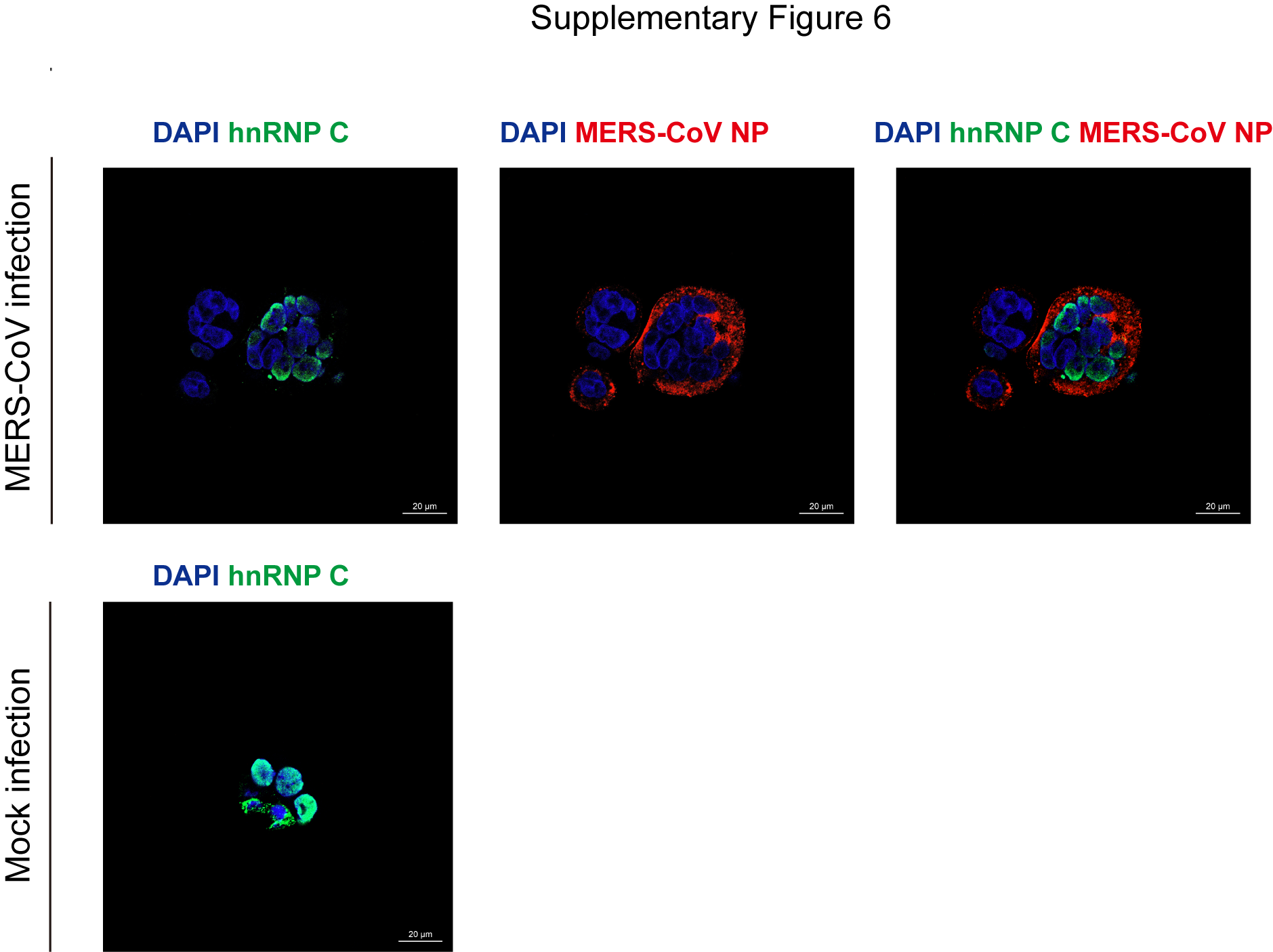

Supplement: Supplemental Material [file TEMI_A_2032372_SM2191.zip › Suppl files/Supplementary Figure 6.png]
